# Echocardiography Screening for Latent Rheumatic Heart Disease: What Can We Do in Indonesia?

**DOI:** 10.3389/fsurg.2020.00046

**Published:** 2020-08-19

**Authors:** Amiliana M. Soesanto, Luh Oliva Saraswati Suastika

**Affiliations:** ^1^Department of Cardiology and Vascular Medicine, Faculty of Medicine, Universitas Indonesia/National Cardiovascular Center Harapan Kita, Jakarta, Indonesia; ^2^Department of Cardiology and Vascular Medicine, Faculty of Medicine, Udayana University, Udayana University Hospital, Denpasar, Indonesia

**Keywords:** rheumatic heart disease, latent, screening, echocardiography, Indonesia

## Abstract

Rheumatic heart disease (RHD), a sequela of acute rheumatic fever (ARF), is a preventable disease but remains a significant health problem, especially in developing countries. It causes disability, poor quality of life, early mortality, and national economic burden. The World Heart Federation (WHF) aimed to achieve a 25% reduction in premature deaths from ARF and RHD among individuals aged <25 years by 2025. Primordial and primary prophylaxis of RHD is aimed to prevent the occurrence of ARF, while the goal of secondary and tertiary prophylaxis is to limit the progression and reduce the consequences of RHD. Early recognition of RHD is important for early prophylaxis strategies to inhibit any progression to advanced stages. In 2012, WHF introduced the latest echocardiographic criteria to recognize the early stage of RHD. This includes the evaluation of pathological regurgitation jet and morphological features of RHD based on 2D, color, and spectral Doppler criteria. In remote areas, portable echocardiography is preferable for RHD screening. Previous portable devices were only capable of producing 2D and color images. Hence, a simplified echocardiographic criterion without spectral Doppler evaluation is needed in selected areas. Indonesia is a developing country, an archipelago with a population of over 250 million. Currently, there are no data on ARF incidence and RHD prevalence nationwide. The only data available are the number of patients in advanced stages who came to referral centers for further management. The screening program has to be introduced in Indonesia as part of national RHD prophylaxis.

## Introduction

Rheumatic heart disease (RHD) is a sequela of acute rheumatic fever (ARF). The disease is preventable, but it remains a significant health problem, especially in developing countries. It may cause disability, poor quality of life, early mortality, and national economic burden. A systematic review and meta-analysis of active surveillance studies found that in general, the prevalence of RHD was 2.9 per 1,000 people (95% CI: 1.7–5.0) if detected by cardiac auscultation and 12.9 per 1,000 people (95% CI: 8.9–18.6) by echocardiography (1). A study from New Zealand collecting ARF and RHD cases in 2000–2009 reported a mean annual mortality rate of 4.4 per 100,000 (95% CI: 4.2–4.7) and an average annual diagnosis-related group (DRG)-based cost of hospital admissions of $12.0 million (95% CI: $11.1–12.8).

The World Heart Federation (WHF) aimed to achieve a 25% reduction in premature deaths from ARF and RHD among individuals aged <25 years by the year 2025. Early recognition of RHD is essential for early prophylaxis strategy to inhibit any progression to advanced stages. This paper would like to discuss a proposed strategy for conducting effective screening for latent RHD in Indonesia as a developing country with challenging coverage areas and limited resources.

## Subsection

### Natural History of RHD and Prophylaxis Strategy

RHD is the long-term consequence of ARF, which is caused by an autoimmune reaction to group A beta-hemolytic streptococcal (GAS) infection. The predisposing factor includes poor hygiene, crowded household, and lack of access to proper medical services, commonly found in the middle- and low-income population (2). There are theories of the pathogenesis of ARF. The most popular theory is an occurrence of cross-reactivity between the heart tissue and GAS through molecular mimicry, where foreign antigen resembles host tissues (3). When the host immune response targets the infecting organism, both the infecting organism and host cells are attacked. In ARF, the M protein on GAS cell membrane is the foreign antigen (4). It cross-reacts with myosin and induces T-cell mediated attack of cardiac tissue. When the cardiac tissue is damaged, antibodies activate the valve endothelium to express adhesion molecules and allow T cells to be activated by GAS to invade the valve tissue and lead to autoantibodies that attack valve tissue (5). This leads to further inflammation of heart valves, and the recurrent episodes of infection lead to fibrosis over time, which then develops as RHD (6). All heart valves are equally susceptible to this pathologic process. However, mitral valve (MV) is predominantly involved in rheumatic carditis without clear explanation (7).

Latent RHD occurs in asymptomatic patients with echocardiographic evidence of RHD without the history of ARF. RHD is usually clinically silent until it manifests during adulthood. Adult patients are frequently admitted to the hospital with worse complications of RHD, including heart failure, pulmonary hypertension, and atrial fibrillation. Screening for latent RHD is useful in recognizing the disease progression rate and detecting people who might benefit from secondary prophylaxis.

Cohort studies evaluating natural history could estimate the progression rate of latent RHD. The progression rate could be affected by continuing inflammatory response from the first and recurrent GAS infection and ARF episodes. Latent RHD in children has variable disease outcomes ranging from regression to normal in half of the cases to persistence and progression of symptoms in the remaining cases (8–10), and it continues over time (2). Children with borderline and mild definite RHD have better outcomes but remain at risk of disease progression (9). Even minor echocardiographic changes defined as borderline RHD and other non-specific valve abnormalities were reported as an indication of increased risk of progression and development of definite RHD (11). Advanced disease, morphological rheumatic MV features, and younger age in this population were risk factors for worse outcome (9).

It is considered that early application of secondary prophylaxis in individuals with latent RHD may alter disease progression. Unfortunately, this hypothesis has not been established in any prospective study or randomized control trial. An earlier study reported that intramuscular regular benzathine penicillin might cause regression of regurgitation murmur and prevent the development of stenosis in post rheumatic fever patients at 5–10 years' follow-up (12). Other studies reported that initiating the secondary prophylaxis in earlier stages of RHD, including latent RHD may potentially prevent progression to definite RHD (8, 9, 13). Most of the studies were relatively small and followed the natural history of the disease. A randomized trial is required to determine the effect of secondary prophylaxis initiating in latent RHD to alter the disease course. For the time being, perhaps the decision to initiate secondary prophylaxis at diagnosis would be based on the risk of disease progression.

### Echocardiographic Features of RHD

RHD predominantly affects the left-sided cardiac valves with various morphological changes. Regurgitation lesion dominates in the young population, while mixed or stenotic lesion dominates in more advanced age. The morphological features of RHD of the MV include thickening of the tips of the MV leaflets, chordal thickening, excessive leaflet tip motion, and restricted leaflet motion. Valvular thickening is most pronounced at the leaflet tips. After a longer period, a chronic progression of RHD may lead to shortening and fusion of the chordae, leaflet thickening, and calcification or commissural fusion, which are the typical feature of an advanced stage of RHD. It should be assessed in diastole, giving the appearance of a “hockey stick” if the anterior leaflet is affected or the posterior MV leaflet is immobile, which is the hallmark of RHD (14).

Although the aortic valve (AV) is commonly affected, it is rarely isolated and usually accompanied by concomitant MV involvement. Unlike the RHD feature of MV, echocardiographic features of RHD in AV are not specific to RHD. The focal thickening of AV commonly occurs at the free edge of AV and is assessed subjectively. The nodal or irregular thickening of leaflet edges causes leaflet retraction, which leads to coaptation defect and aortic regurgitation (AR). Restricted leaflet motion is the predominant mechanism of rheumatic AR. Commissural fusion and thickening of AV leaflets are more prevalent in advancing age. Prolapse of the AV leaflet is not a specific feature of rheumatic aortic lesions, and other etiologies should be excluded (14).

#### The WHF Echocardiography Criteria

In 2012, the WHF published consensus-based criteria for echocardiography diagnosis of RHD (Table 1). Three categories are defined based on 2D, color Doppler, and continuous-wave echocardiography assessment, which includes definite RHD, borderline RHD, and normal. Criteria for pathological mitral regurgitation (MR) and AR and rheumatic morphological changes of both MV and AV were also defined in the guideline (14).

**Table 1:** The World Heart Federation criteria for diagnosing RHD (14).

**Definite RHD (either A, B, C, or D)**	**Definite RHD (either A, B, C, or D)**
**Age ≤ 20 years**	**Age > 20 years**
A. Pathological MR and at least two morphological features of RHD of the MV	A. Pathological MR and at least two morphological features of RHD of the MV
B. MS mean gradient ≥ 4 mmHg	B. MS mean gradient ≥ 4 mmHg
C. Pathological AR and at least two morphological features of RHD of the AV	C. Pathological AR and at least two morphological features of RHD of the AV
D. Borderline disease of both the MV and AV	D. Borderline disease of both the MV and AV
**Borderline RHD (either A, B, or C)**	**Borderline Not Applicable to Those Age >20 Years**
A. At least 2 morphological features of RHD of the MV without pathological MR or MS	
B. Pathological MR	
C. Pathological AR	
**Pathological MR**	**Pathological AR**
Seen in two views	Seen in two views
In at least one view, jet length ≥ 2 cm	In at least one view, jet length ≥ 1 cm
Velocity ≥ 3 m/s for one complete envelope	Velocity ≥ 3 m/s in early diastole
Pan-systolic jet in at least one envelope	Pan-systolic jet in at least one envelope
**MV Morphological Features**	**AV Morphological Features**
AMVL thickening ≥ 3 mm (age ≤ 20 years), ≥4 mm (age 21–40 years), ≥5 mm (age > 40 years)	Irregular or focal thickening
Chordal thickening	Coaptation defect
Restricted leaflet motion	Restricted leaflet motion
Excessive leaflet tip motion during systole	Leaflet prolapse

*RHD, rheumatic heart disease; MR, mitral regurgitation; MV, mitral valve; MS, mitral stenosis; AR, aortic regurgitation; AV, aortic valve; AMVL, anterior mitral valve leaflet*.

#### Simplified Criteria

The WHF 2012 echocardiographic criteria of RHD have been used as a reference for a screening of RHD worldwide. However, it could be challenging in remote areas where there is a lack of advanced tools to evaluate Doppler and skilled practitioners who can examine all the complex criteria rapidly. Several studies had introduced simplified echocardiography criteria for RHD screening, mainly focusing on the MV. This approach seemed to be reasonable since the MV is the most common valve involved. Some RHD screening in several areas use a single parameter of MR jet length, regardless of any morphological changes (15, 16). When compared with reference criteria, the simplified approach yielded good accuracy.

While simplified echocardiography screening assessing MR jet seems feasible and allows rapid screening of RHD, diastolic leaflet restriction is considered the principal finding of RHD; thus, assessing the combination of regurgitation jet and morphological changes is recommended. When developing simplified screening criteria, both the functional and morphological features of the valve should be considered to avoid oversimplifying the case detection. A simple scoring system for RHD diagnosis had been introduced by Nunes et al. (10). They did three phase studies, including (1) derivation of simplified criteria, which included school children in Brazil; (2) validation of the new simplified criteria, which included school children in Uganda; and (3) outcome prediction with median follow-up length of 2.3 years.

The approach is more practical, especially when it can be performed using a non-spectral Doppler handheld echocardiography. The components included in the scoring system are MV anterior leaflet thickening, excessive leaflet tip motion, regurgitation jet length ≥2 cm, AV focal thickening, and any AR, resulting in a total score of 0 to 21 points (Table 2). According to the predicted probability, low-risk scores were 0 to 6, intermediate-risk scores were 7 to 9, and high-risk scores were ≥10. The low-risk total score estimated 1% probability of definite RHD, while intermediate and high scores would predict 15.7 and 57% probability, respectively.

**Table 2:** Simplified echocardiography scoring system (10).

**Variable**	**β-coefficient**	**SE**	***Z* value**	***p*-value**	**Points**
**Mitral Valve**					
Anterior leaflet thickening	2.941	0.597	4.922	<0.0001	3
Excessive leaflet tip motion	3.102	0.543	5.716	<0.0001	3
Regurgitation jet length ≥ 2 cm	5.601	0.705	7.941	<0.0001	6
**Aortic Valve**					
Irregular focal thickening	4.460	0.970	4.597	<0.0001	4
Any regurgitation	4.794	0.718	6.679	<0.0001	5

*SE, standard error*.

This scoring system is the first one developed for risk stratification in latent RHD. A total score of ≤ 6 is considered as low risk, with a predicted disease progression of ≤ 5.6%. Medium risk score was 7–9, which predicted 8.3–17.5% progression, and high-risk score was ≥10 for predicting disease progression of ≥24.6%. Another prospective cohort study validated this scoring system in subclinical RHD schoolchildren as part of an RHD screening program in Brazil. They reported that the simplified echo study was accurate in predicting an early outcome with 29 ± 9 (range 11–48) months' follow-up (17).

## Discussion

### The Problem of RHD in Indonesia

Indonesia is the largest archipelago in the world, with more than 17,000 islands and an area of 1,904,569 km^2^. It is the world's fourth most populated country, with a population of over 267 million. Currently, there are no official data regarding ARF incidence and RHD prevalence nationwide. Studies about this are so limited. This might be due to challenging transportation to many remote areas and islands, lack of logistics for the screening, and limited healthcare workers to cover a large screening area. The Asian data pooled from 17 reports showed that 28.0 per 1,000 people (95% CI: 16.6–49.9) were affected by the disease; none of the reports came from Indonesia (1). From the worldwide epidemiology data of acute rheumatic disease and RHD, the only data available from Indonesia reported that the prevalence of RHD was <1% from 1970 through 1990 (18). However, some recent studies reported local prevalence of RHD in some parts of Indonesia (19–21).

A study reported a pattern of RHD cohort from a mining population in Papua Province, Indonesia. The total number of RHD patients identified from medical records reviewed and included in this study was 83 out of 15,608 workers. RHD incidence density was 6.84 per 10,000 person-years at follow-up. Among cases, mitral stenosis was the most common valvular lesion at initial presentation (41.0%), and 6.0% were multivalvular (19). An echocardiography study from a cardiac center in Bandung, West Java, Indonesia, found that during 13 months of observation, a total of 108/4,682 (2.3%) of patients showed echocardiography morphology of RHD, with a mean age of 44 years (17–75) and predominantly female (78%). A combination of MV and AV involvement was the most common echo findings (56.48%), followed by a mixed MV disease in 25.92%, whereas isolated mitral stenosis was found in 17.6% patients (20). Just recently, Suastika et al. conducted a local RHD screening in one remote area in Bali (21). Schoolchildren (mean age 13.9 years) were screened in school using tablet echocardiography according to the WHF criteria. This region was selected based on a high referral pattern of adult RHD cases from a particular area. There were only three borderline RHD and no definite RHD cases.

The observed RHD prevalence in schoolchildren in Bali in one remote area was lower than expected, although the area was chosen for screening due to high adult RHD cases admitted to the hospital. This may be due to recent improvements in the quality of life and better access to healthcare (21). However, in Papua, RHD prevalence was also lower than the worldwide prevalence (0.068%), maybe because the study population was adult patients admitted to the hospital (19). In Bandung, the prevalence was quite high, presumably because the study was performed in a cardiac referral center with cardiac patients as the population. The different results from small studies in Indonesia urge the need to perform screening in the general population to determine the real prevalence in Indonesia (20).

Apart from the lack of a nationwide epidemiology data, the fact that RHD causes social and economic burden for our country is undeniable. An unpublished valvular registry from the National Cardiovascular Center Harapan Kita, Jakarta, Indonesia, showed that during the years of 2016–2019, there were 7,112 valvular cases, and 40.5% of them were of rheumatic etiology. Since the center is the highest national reference hospital in Indonesia, the admitted cases were mostly severe and needed further intervention, either surgical or non-surgical. This is just the tip of the iceberg, and there must be more undetected cases spread globally. Screening for latent RHD is the earliest step to obtain epidemiologic data on the disease, which can be used for a further strategy to eradicate RHD in the future. Indonesia is a developing country with limited financial resources, so the cost-effectiveness of the screening should be carefully studied before organizing an effective and efficient nationwide RHD screening program.

### What Can We Do in Indonesia?

A good collaboration between related nationwide physicians and a strategic plan is essential to run the program successfully. How to diagnose, what device should be used, who performs the screening, whom the target will be, and a financial budget should be taken into consideration before starting the screening program. Affordable and accurate screening tests for RHD are needed.

The Jones criterion has been a well-established criterion for diagnosing ARF. On the other hand, the diagnosis of RHD is determined by a combination of clinical, echocardiographic features, and any history of ARF. It is known that echocardiography screening is more sensitive than cardiac auscultation for detecting valvular lesions, especially in subclinical RHD. As explained above, the 2012 WHF criteria are a consensus-based criteria for the echocardiographic diagnosis of RHD (14). The simplified echocardiography scoring system to diagnose RHD introduced by Nunes et al. offered a more practical and simpler criterion and is suitable for handheld echocardiography application (10). The accuracy of echocardiographic examination even by a handheld echocardiography tool was significantly better than cardiac auscultation alone (1, 16, 22, 23). Most of the RHD screening programs utilized portable echocardiography for more comfortable mobility in remote areas, and in general, they showed a reasonable value for RHD screening. Many studies reported the prevalence of RHD and RHD screening, mainly in regions with a high prevalence of RHD, including Uganda (24, 25), Sudan (26), South Africa (27), Ethiopia (27), Italy (28), Brazil (29), Egypt (30), and Fiji (31). In general, these studies utilized standard portable echocardiography or handheld machines with simplified criteria for on-site screening. The device significantly improves RHD's detection and may be a cost-effective screening strategy for RHD in resource-limited settings. It has been presented as a promising alternative to standard echocardiography in a remote setting, and perhaps utilizing these simplified criteria with handheld echocardiography will be a good option for the RHD screening program in Indonesia as more areas can be reached; hence, more cases can be detected.

Another essential issue is competent screening operators to make accurate detection of latent RHD. In Indonesia, there are only approximately 1,200 cardiologists and less than a 100 sonographers for serving more than 260 million population. The limited number of cardiologists and sonographers to perform and interpret echocardiograms is one of the significant challenges. A task changing screening to non-expert health workers might overcome this problem. Optimal training for nurses, general physicians, or other medical staff should be conducted to achieve a reliable screening result. Some studies reported good echocardiography result performed by briefly trained non-expert individuals (31–33). An RHD screening in Uganda performed by nurses trained with handheld echocardiography had reasonable sensitivity and specificity (33). In Fiji, after an 8 week training program, nurses could perform focused ultrasound screening, which had high diagnostic accuracy (0.89) with reasonable sensitivity and specificity (31). The strategy of training non-experts to perform RHD screening may be considered as a way to expand the screening program to cover more remote areas. However, a good network will be needed among cardiologists, physicians, and other healthcare providers involved in the screening program to guarantee an accurate result.

Our last concern is deciding whom to screen and which area should be focused on for screening. The peak incidence of ARF is in school-aged children (aged 5–14 years). Screening RHD in high-prevalence areas is recommended to detect people who might benefit from secondary prophylaxis. In most echocardiographic screening surveys to date, this group has been targeted. The screening studies were done through the school screening program (9, 25, 29, 34–36). Other than the age group, the targeted population will help in better screening results. RHD results from a complex interaction among the host, agent, and environment. It is known that there is genetic susceptibility in RHD, and relatives of children with RHD belong to the high-risk group. Given genetic susceptibility and a shared environment, siblings of RHD-positive cases are more likely to have definite RHD. In Uganda, a study by Aliku et al. showed that siblings of children with RHD were more likely to have definite RHD; the highest relative risk was in children with definite RHD. Sibling screening should be considered in the RHD screening program following the detection of definite RHD cases (24). High-prevalence areas could be suspected based on the origin of the RHD patients referred to the hospital. Collaboration with the local physicians, local healthcare centers, and local schools is useful to define the targeted area for screening.

## Summary

In Indonesia, the RHD screening program has not yet developed nationwide. The cost of diagnostic tools and the requirement of trained personnel make the accurate detection of RHD remain a challenge. Developing an effective and efficient RHD screening program will enable us to increase the coverage area and reach a more screened population. The simplified criteria and the use of handheld echocardiography will help the screening program to cover a larger endemic area where there is a shortage of logistic and skilled physicians. Targeting to screen schoolchildren and siblings from where referral cases originated could be an ideal and feasible strategy for the screening program.

## Author Contributions

AS: the idea, literature searching, writing the manuscript, and review/finalization. LS: literature searching and writing the manuscript. All authors: contributed to the article and approved the submitted version.

## Conflict of Interest

The authors declare that the research was conducted in the absence of any commercial or financial relationships that could be construed as a potential conflict of interest.
